# Genotype-Phenotype Correlation of 2q37 Deletions Including *NPPC* Gene Associated with Skeletal Malformations

**DOI:** 10.1371/journal.pone.0066048

**Published:** 2013-06-21

**Authors:** Elisa Tassano, Jens Buttgereit, Michael Bader, Margherita Lerone, Maria Teresa Divizia, Renata Bocciardi, Flavia Napoli, Giovanna Pala, Frédérique Sloan-Béna, Stefania Gimelli, Giorgio Gimelli

**Affiliations:** 1 Laboratorio di Citogenetica, Istituto G. Gaslini, Genova, Italy; 2 Experimental and Clinical Research Center (ECRC), a joint institution of the Max Delbrück Center for Molecular Medicine (MDC) and the Charité Medical Faculty, Berlin, Germany; 3 Max Delbrück Center for Molecular Medicine (MDC), Campus Berlin-Buch, Berlin, Germany; 4 Laboratorio di Genetica Molecolare, Istituto G. Gaslini, Genova, Italy; 5 Dipartimento di Neuroscienze, Riabilitazione, Oftalmologia, Genetica e Scienze Materno-Infantili, (DiNOGMI), and Laboratorio di Genetica Molecolare, Istituto G. Gaslini, Genova, Italy; 6 Service of Genetic Medicine, University Hospitals of Geneva, Geneva, Switzerland; 7 Dipartimento di Pediatria, Istituto G. Gaslini, Università di Genova, Genova, Italy; Baylor College of Medicine, United States of America

## Abstract

Coordinated bone growth is controlled by numerous mechanisms which are only partially understood because of the involvement of many hormones and local regulators. The C-type Natriuretic Peptide (CNP), encoded by *NPPC* gene located on chromosome 2q37.1, is a molecule that regulates endochondral ossification of the cartilaginous growth plate and influences longitudinal bone growth. Two independent studies have described three patients with a Marfan-like phenotype presenting a *de novo* balanced translocation involving the same chromosomal region 2q37.1 and overexpression of NPPC. We report on two partially overlapping interstitial 2q37 deletions identified by array CGH. The two patients showed opposite phenotypes characterized by short stature and skeletal overgrowth, respectively. The patient with short stature presented a 2q37 deletion causing the loss of one copy of the *NPPC* gene and the truncation of the *DIS3L2* gene with normal CNP plasma concentration. The deletion identified in the patient with a Marfan-like phenotype interrupted the *DIS3L2* gene without involving the *NPPC* gene. In addition, a strongly elevated CNP plasma concentration was found in this patient. A possible role of *NPPC* as causative of the two opposite phenotypes is discussed in this study.

## Introduction

During embryonic development and in postnatal life, longitudinal bone growth occurs at the cartilaginous growth plates located at the ends of the vertebrae and long bones. This process involves systemic hormones and local regulators that are finely tuned to allow harmonious and coordinated bone growth. Among these, CNP (C-type natriuretic peptide) plays an essential role in regulating bone growth by influencing cartilage homeostasis, proliferation and differentiation of osteoblasts and osteoclasts, and endochondral bone growth [1]–[4]. It is encoded by the *NPPC* gene located on chromosome 2q37.1 and exerts its effect through the interaction with the natriuretic peptide receptor B (NPR2 or NPRB) [5]. Knock-out mice for the CNP coding gene showed marked dwarfism due to impaired endochondral ossification [6]–[8].Targeted expression of CNP in chondrocytes rescued the dwarfism of CNP deficient mice, indicating that CNP acts as a local regulator of longitudinal bone growth [7]. Up to now no mutation or loss of the *NPPC* gene associated with dwarfism has been reported in humans. Homozygous mutations of the *NPR2* gene have been identified in patients affected by acromesomelic dysplasia or Maroteaux type (MIM 602875), while heterozygous mutations of the same receptor gene have been associated with short stature [9]–[10]. Conversely, overexpression of the *NPPC* gene determines skeletal overgrowth in transgenic mice [3] and in humans [11]–[13]. A recent genome-wide association study suggested that variation in the CNP signaling pathway, involving the *NPPC* and *NPR3* genes, plays an important role in determining human body height [14].

We report on two different patients with *de novo* 2q37 deletions. Patient 1 is affected by a pathological short stature and presents a 2q37.1 deletion of 1.9 Mb, while Patient 2 has a 2q37.1q37.3 deletion of 4.5 Mb associated with a Marfan-like phenotype. We discuss the possible role of *NPPC* in determining the two opposite phenotypes.

## Results

### Clinical Report

#### Patient 1

The patient, a 7 year-old-girl, is the second child of healthy non-consanguineous parents; parents' height was normal (father's height was 180,8 cm and mother's height 157,4 cm). In the family, a maternal first cousin had severe mental delay with abnormal behaviour and a paternal granduncle showed bilateral upper limb malformation. The patient has a healthy 9-year-old sister. Ultrasound examination revealed intrauterine growth retardation and polyhydramnios. The patient was born at 37 weeks of gestation by spontaneous delivery.

**Figure 1:**
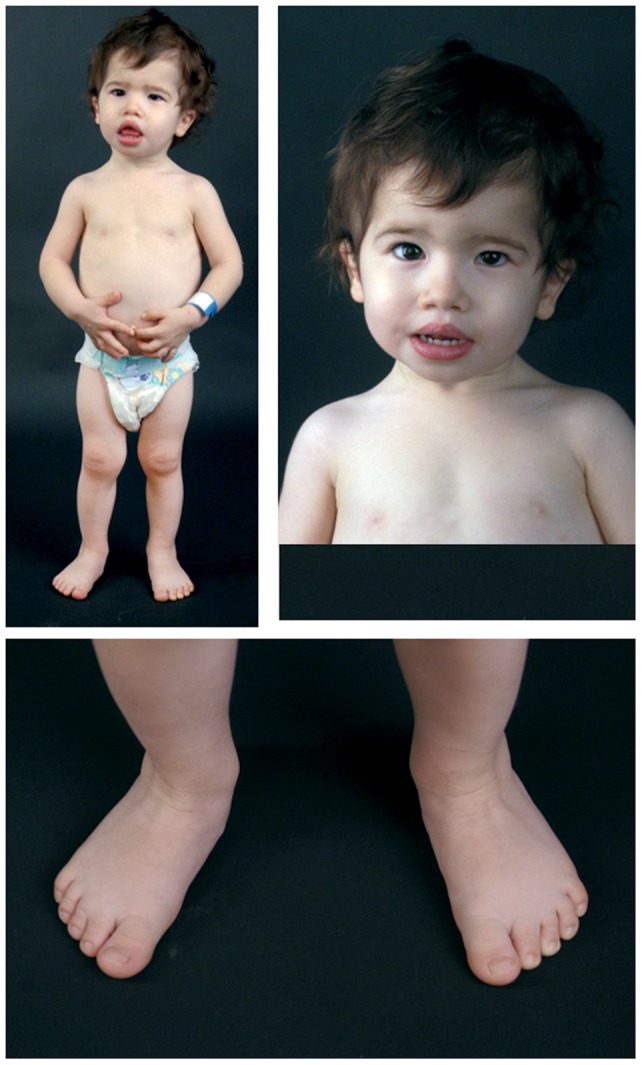
Clinical signs of the patient 1. The patient 1 at 3 years of age: short stature, prominent forehead, strabismus, epicanthus, low-set-ears, depressed nasal bridge, short phyltrum, V-shaped mouth. Mild brachydactyly, cutaneous syndactyly of 2nd and 3rd toes.

Birth weight was 2275 g (<3^th^ centile), length 42 cm (<3^th^ centile) and the occipitofrontal circumference (OFC) 34.5 cm (75^th^ centile); Apgar score was 9 and 10 at 1 and 5 minutes, respectively. At the age of 20 months, growth parameters were weight 7620 g (<3^th^ centile), height 73 cm (<3^th^ centile), OFC 46.5 cm (50^th^–75^th^ centile). Clinical examination showed facial dysmorphisms including pyriform skull with prominent forehead, upward slanting eyebrows, strabismus, epicanthus, low-set ears, depressed nasal bridge, short philtrum, V-shaped mouth and short upper lip. Feet showed mild brachydactyly, cutaneous syndactyly of 2^nd^ and 3^rd^ toes bilaterally, axial and limb girdle hypotonia, mild lower limb dysmetria (left>right), joint laxity. Growth has always been quite poor. At 6 years 5 months height was 103.4 cm (3^th^ centile). Psychomotor and language development were delayed. Ophthalmological examination showed refractive errors and, at the age of two months, peripheral hearing loss was confirmed by auditory brainstem response (ABR test). Chest X-ray revealed 13 rib pairs and brain MRI showed a corpus callosum dysgenesis. Echocardiogram showed mild mitral valve insufficiency. Arginine stimulation test showed a normal GH response, thus ruling out GH deficiency. Photographs of the patient is shown in Figure 1.

#### Patient 2

**Figure 2:**
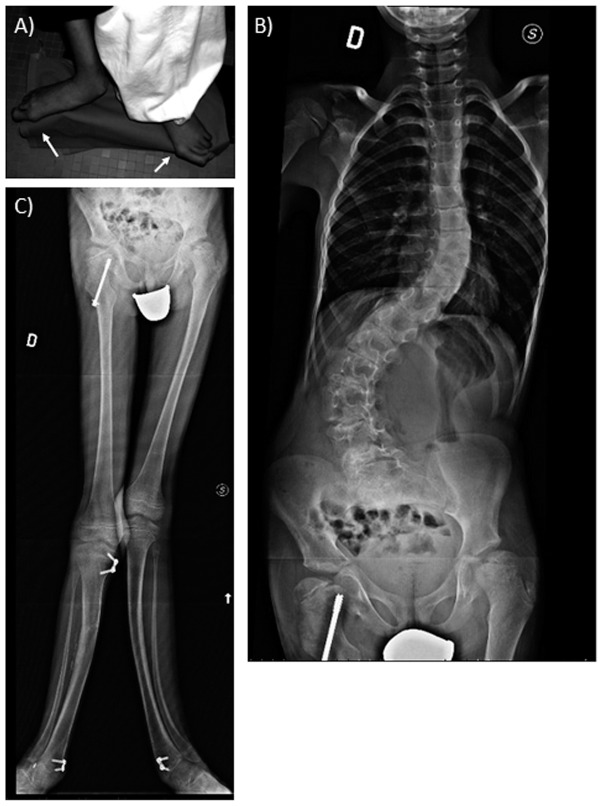
Clinical signs of the patient 2. A) Details of feet, showing very long hallux bilaterally B) X-rays, showing severe scoliosis. C) Long and curved lower limbs.

The patient is a 12-year-old boy born to non-consanguineous parents. No family history of congenital anomalies or mental retardation was reported. He has a healthy 4 year-old sister. The patient was born at full term after an uneventful pregnancy by spontaneous vaginal delivery. Exposure to drugs or infection during the pregnancy was denied. At birth, weight was 3.300 kg (25^th^–50^th^ centile), length 54 cm (90–95^th^ centile) and occipitofrontal circumference (OFC) 34 cm (10^th^–25^th^ centile). Psychomotor development was normal. At the age of 12 years, weight was 30 kg (5^th^ centile), height 165 cm (>97^th^ centile) and OFC 53.7 cm (>50^th^ centile). Marfanoid habitus, arachnodactyly of hands and feet with very long hallux bilaterally associated and mild syndactyly of 3^rd^, 4^th^ and 5^th^ fingers, and severe scoliosis were observed (Fig. 2). Echocardiography showed patent ductus arteriosus.

### Cytogenetic and Array-CGH Analysis

Cytogenetic analysis, performed on GTG-banded metaphases from cultured lymphocytes of both patients and their parents showed normal karyotypes. Considering the phenotypic abnormalities of the patients, array CGH analysis (Agilent G3 400 K) was performed.

**Figure 3:**
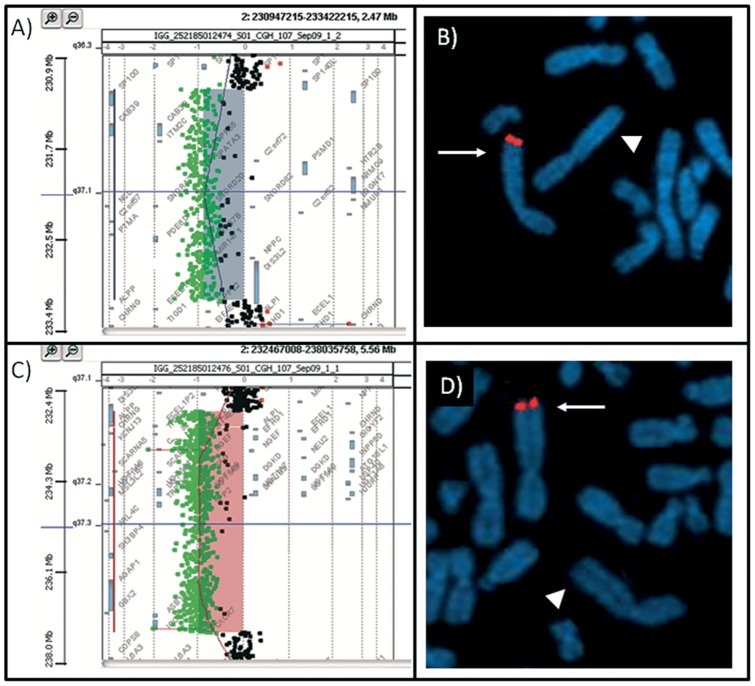
Array-CGH and FISH results. A) Result of array-CGH analysis of chromosome 2 with Agilent Human Genome CGH microarray Kit G3 400 in patient 1. The 1.914 Mb interstitial q37.1 deleted region of chromosome 2 extends between oligomers A_18_P13670199 (231,264,956 bp) and A_16_P00618306 (233,178,325 bp) flanked by oligomers A_16_P00615757 (231,257,468 bp) and A_16_P00618312 (233,181,399 bp) (UCSC Genome Browser, http://genome.ucsc.edu/; February 2009). B) FISH with BAC clones RP11-395A23 (AC010149) (chr2:231,304,236–231,476,367). The arrowhead indicates the deleted chromosome 2. D) Result of array-CGH analysis of chromosome 2 with Agilent Human Genome CGH microarray Kit G3 400 in patient 2. The 4.515 Mb interstitial deletion at bands q37.1q37.3 of chromosome 2 was comprised between oligomers A_16_P16076619 (232,963,736 bp) and A_16_P36124457(237,479,062 bp) flanked by oligomers A_16_P16076610 (232,954,321 bp) and A_16_P36124475 (237,483,914 bp). C) FISH with RP11-485M18 (AC079400)(chr2:236,766,818-236,919,215). The arrowhead shows the deleted chromosome 2.

In patient 1 array CGH analysis revealed a *de novo* interstitial deletion of ∼1.9 Mb at 2q37.1 band (chr2:231,264,596–233,178,325 )×1 (Fig. 3, A). The presence of the deletion was confirmed by FISH with BAC clone RP11-395A23 (AC010149) (Fig. 3, B).

**Figure 4:**
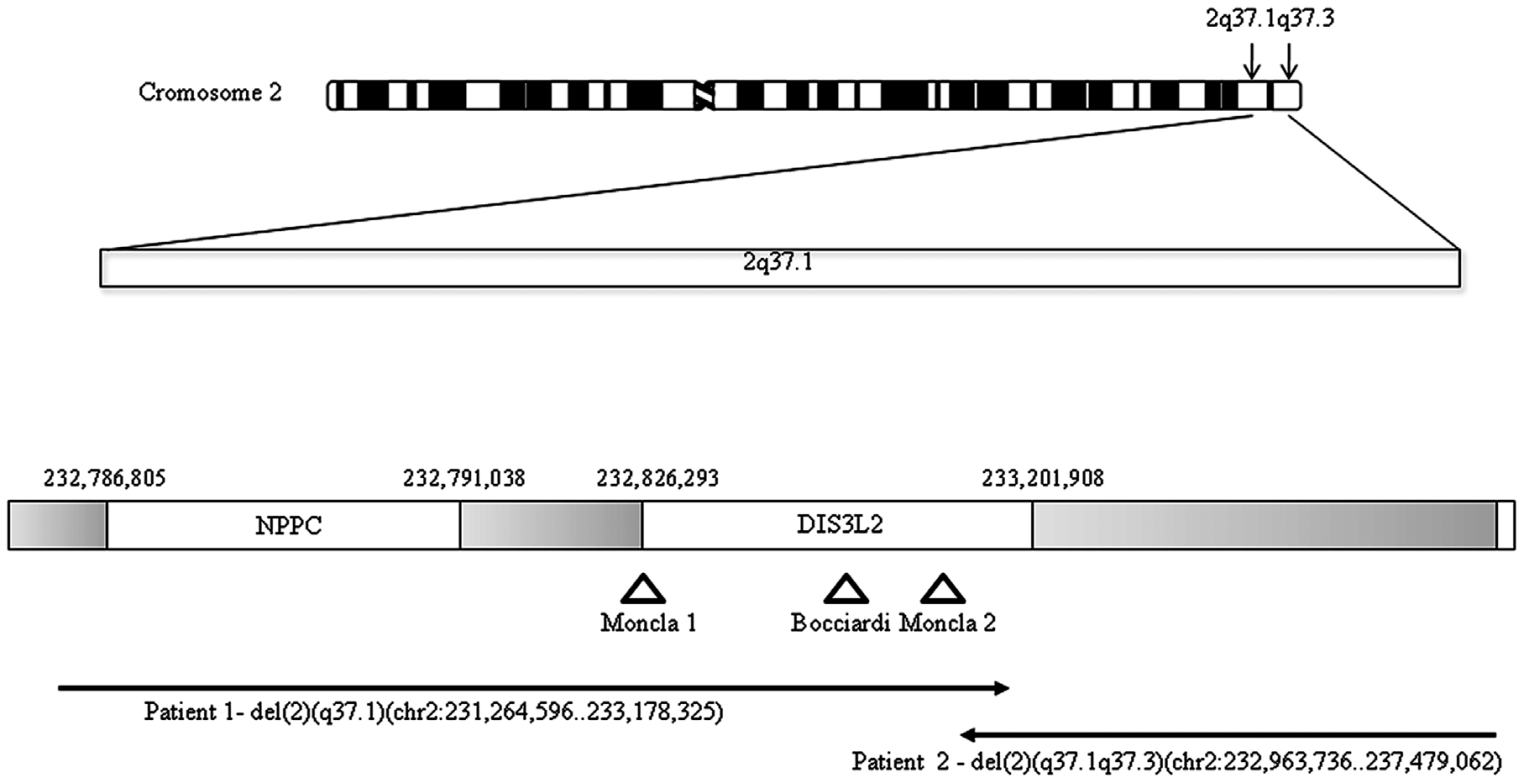
Schematic representation of the genomic region spanning the described deletions according to the UCSC Genome Browser Feb 2009 (GRCh37/hg19). Several annotated genes and ESTs are present, the two deletions are indicated by black arrows (patient 1 and patient 2), whereas triangles indicate the position of the translocation breakpoints previously published [11]–[12].

At least 20 annotated genes were included within the deleted region. Among those genes, the *NPPC* gene had been previously associated with overgrowth and skeletal anomalies [11]–[13]. The deletion also leads to the truncation of *DIS3L2* locus in the intronic region comprised between exons 14 and 15 (Fig. 4).

In patient 2, a *de novo* interstitial 2q37.1q37.3 deletion of ∼4.5 Mb (chr2:232,963,736 -237,479,062)×1 was identified by array CGH (Fig. 3, C). The deletion was confirmed by FISH with BAC clone RP11-485M18 (AC079400)(Fig 3, D). The deleted region contained 45 annotated genes and its proximal (centromeric) breakpoint interrupted the *DIS3L2* gene in intron 6 (Fig 4).

The two rearrangements shared an overlapping region of 214 kb containing 8 exons of *DIS3L2 l*ocus (from exon 7 to exon 14) and a gene coding for hsa-miR-562 (Fig 4). The microRNA sequence was within intron 9 of *DIS3L2* and it was deleted in both patients. No additional imbalance was detected in patients 1 and 2.

### Gene expression analysis

**Figure 5:**
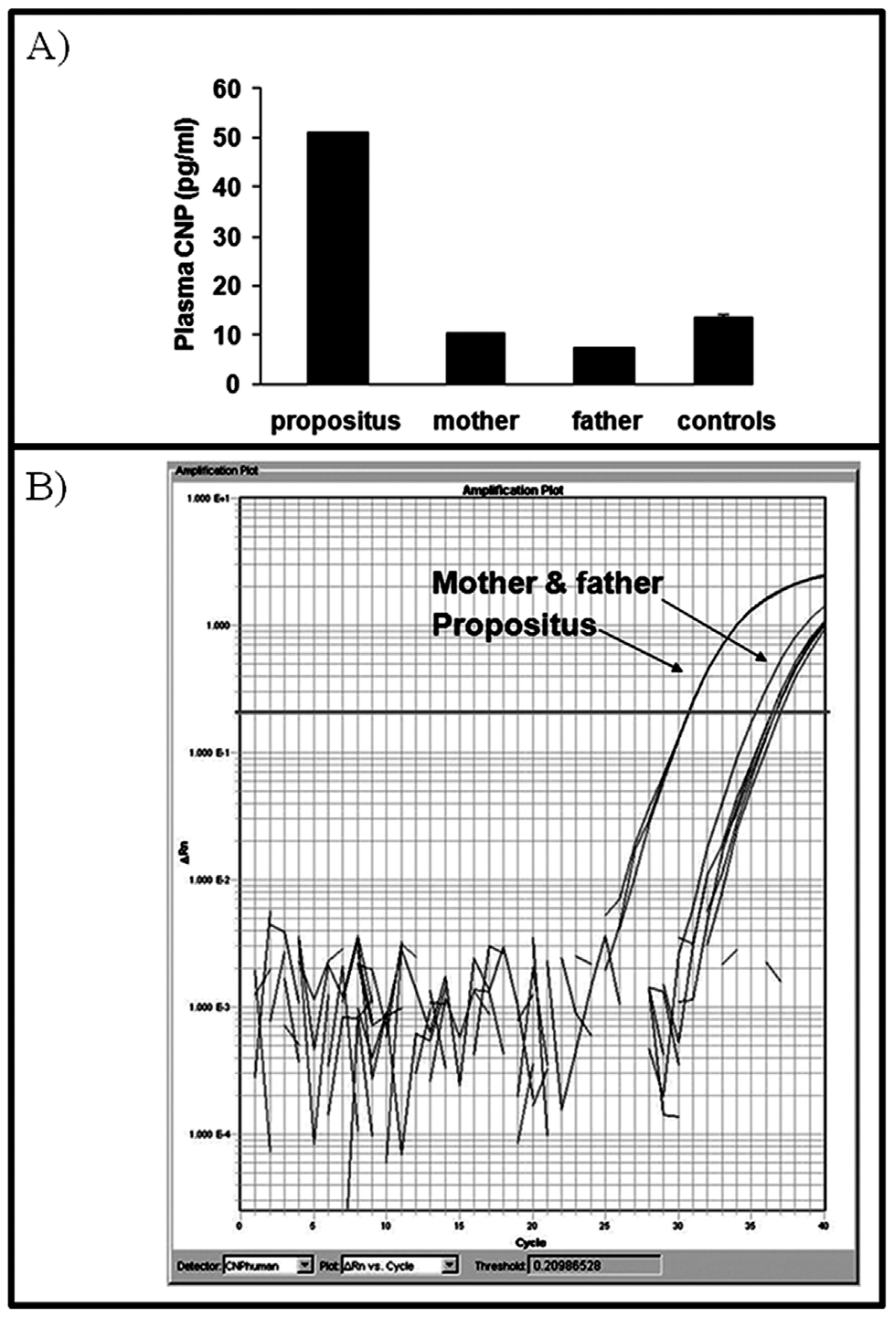
Expression and biochemical results. A) CNP plasma concentration in patient 2, his parents and three controls. The concentration of the peptide was increased more than 4-fold in the propositus compared to parents and unrelated controls (n = 3). B) Analysis of NPPC expression by real-time PCR analysis in lymphoblasts from patient and his parents. The expression of NPPC in the propositus was remarkably higher compared to those of his father and mother.

**Figure 6:**
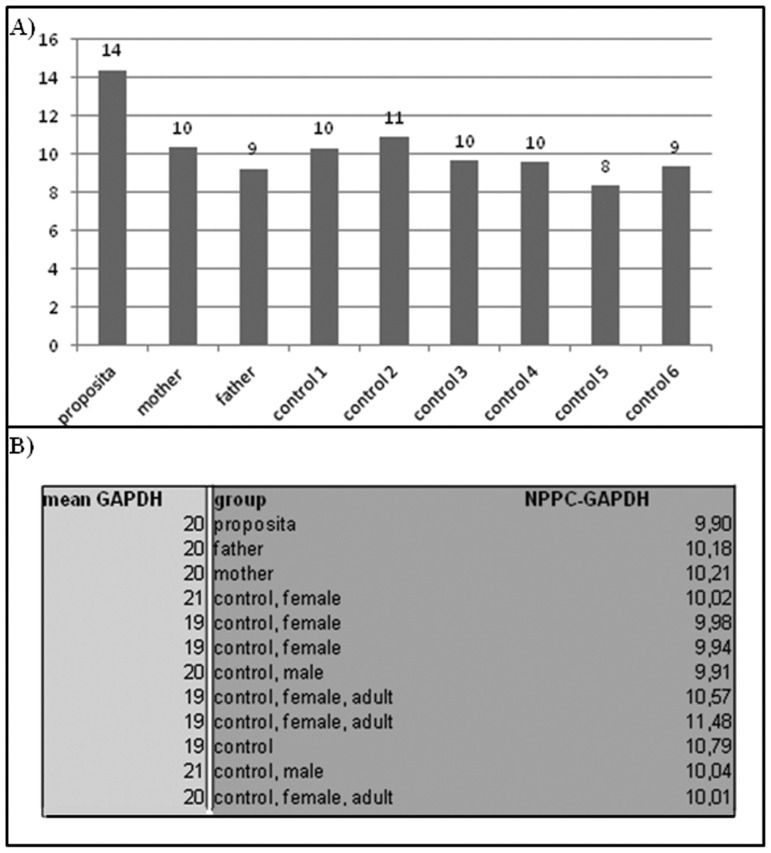
Expression and biochemical results. A) CNP plasma concentration in patient 1, his parents and six controls. The concentration of the peptide was normal compared to parents and unrelated controls (n = 6). B) Analysis of *NPPC* expression by real-time PCR analysis in lymphoblasts from patient, his parents, and ten controls. The expression of NPPC in the proposita was normal compared to those of his parents and unrelated controls (n = 10).

CNP concentration was measured in plasma samples from the two patients, their parents and six female and six male volunteers. For each family member, two double determinations were performed and, for controls, only one double determination. CNP-22 plasma concentration was significantly elevated in patient 2 compared to controls as showed in Fig. 5, A. In this patient, the increased plasmatic levels of CNP were correlated with overexpression of the corresponding gene at mRNA level, as assessed by RT-qPCR (Fig 5, B). Conversely, patient 1 showed normal CNP-22 concentration and normal expression of *NPPC* mRNA, despite complete deletion of one copy of the gene (Fig. 6). The presence of mutations in the promoter region and in the coding sequence of *NPPC* was excluded for both patients (data not shown).

## Discussion

The control mechanisms that allow for coordinated bone growth throughout the body are only partially understood, due to the involvement of many systemic hormones and local regulators. The *NPPC* gene codes for CNP, a molecule that regulates endochondral ossification of the cartilaginous growth plate and, hence, influences longitudinal bone growth. The CNP/NPR2 system has only recently proved to be an important regulator of human growth. Overexpression of *NPPC* has been shown to cause skeletal overgrowth. On the contrary, disruption of *Nppc* or *Npr-2* genes proved to lead to dwarfism and severe skeletal malformations in mice [7]–[8].

Moreover, two independent studies have described three patients characterized by postnatal skeletal overgrowth, Marfanoid habitus, arachnodactyly with abnormally long halluces, and marked overgrowth of the long bones, carrying *de novo* balanced translocations involving the same chromosomal region in 2q37 and three different chromosome partners [11]–[12]. The three breakpoints mapped around the 5′ end of the *DIS3L2* gene (Fig. 4), however this locus was not considered as a candidate.

Recently, germline homozygous mutations in *DIS3L2* have been associated with Perlman syndrome, a rare overgrowth and cancer susceptibility disorder [15]–[16]. However, considering its wide expression, the *DIS3L2* gene does not represent a convincing candidate for a bone affecting phenotype.

The peculiar skeletal phenotype of the translocated patients has been found to an up-regulation of the *NPPC* gene both at mRNA and at circulating peptide level [11]–[12]. As the gene was not disrupted by the translocations nor mutated in the proximal promoter region or in the coding sequence, it was assumed that rearrangements caused a separation of the *NPPC* gene unit from a negative, cis-acting, long-distance regulatory sequence.

Interestingly, a genome-wide association study on northwestern Europeans found that genetic variants close to the *NPPC* gene were significantly linked with body height [14].

Here we report on two patients with a deletion in 2q37 locus identified by array CGH.

Patient 1 showed short stature, cranial asymmetry, moderate psychomotor delay, and a *de novo* 2q37.1 interstitial deletion encompassing many genes including *NPPC* and the first 14 exons of *DIS3L2*. *NPPC* expression and CNP circulating peptide levels were normal.

Mice with targeted disruption of CNP (*Nppc–*/–) displayed striking narrowing of the growth plate of vertebrae and long bones compared to wild type mice and showed severe dwarfism as a result of impaired endochondral ossification [7]. Similarly, the “long bone abnormality” (*lbab*/*lbab*) mice, mutants due to a spontaneous autosomal recessive hypomorphic mutation in the *Nppc* gene, developed severe dwarfism characterized by short tails and extremities, while heterozygous mice did not differ from wild-type mice. These studies confirmed that haploinsufficiency of the *NPPC* gene does not exist in mice [17]. On the contrary, the effects of haploinsufficiency of this gene are evident in humans which could be due to differences between species or some other unknown mechanism(s). CNP is especially crucial for skeletal growth spurt that occurs in early life, is expressed in the growth plate cartilage, and works as an autocrine/paracrine regulator [17]. In the study by Moncla et al. [12], the *NPPC* gene was not expressed in normal lymphoblasts under normal conditions while it is expressed 6-fold in chondrocytes of his patient P1. We think that “normal” expression of *NPPC* in our patient 1was due to a mild overexpression of the unique gene copy and it could interpreted as an attempt to functional recovery.

The second patient of our study is a 12-year-old boy with marfanoid habitus associated with arachnodactyly of hands and feet, mild syndactyly, and severe scoliosis. He is affected by an interstitial 2q37.1q37.3 deletion of 4.5 Mb leading to overexpression of the NPPC gene. The deletion disrupts the DIS3L2 gene at the level of exon 6. The phenotype is very similar to that of the other three patients previously described [11]–[12]. Interestingly, in two cases (patient 2, reported by Bocciardi [11] and patient 2 reported by Moncla [12]), the breakpoints were located into the DIS3L2 gene, while in patient 1 described by Moncla et al. [12] the translocation breakpoint was about 18 Kb centromeric to DIS3L2.

The overlapping region of 214 kb shared by the two deletions contained an additional gene coding for a microRNA named hsa-miR-562 which is expressed only in kidney and colon and regulates EYA1, a critical gene for renal development. The haploinsufficiency of miR-562 contributes to the etiology of Wilms tumor by promoting deregulation of EYA1 [18].Considering the putative role of miR-562 in sporadic Wilms tumor and its specific expression pattern, we couldn't speculate a possible role of this microRNA in determining the phenotypes observed in our patients.

About the other genes present in the two deletions, we could retain that the ones included in patient 1 deletion may have caused the additional dysmorphisms other than the bone phenotype, while the 45 genes in patient 2 deletion appear to have had no significant effects on the phenotype.

To our knowledge, this is the first report of *NPPC* heterozygous deletion associated with short stature. The description of a second case (patient 2) with an opposite phenotype confirms a correlation between the loss of long range control elements which regulate *NPPC* functional activity and skeletal overgrowth. Moreover, these results highlight the possibility of identifying novel therapeutic strategies for the treatment of dwarfism or skeletal overgrowth.

## Materials and Methods

### Ethics Statement

The current study was performed using peripheral blood of the members of the two families treated at the Istituto Giannina Gaslini, Genova, Italy. The parents of the patients gave written informed consent allowing molecular and genetic studies. We didn't request approval by Review Board of our institution, because our study request only classical and molecular cytogenetic analyses. For cytogenetics analyses are sufficient only written informed consent of the parents (DM 21 dicembre 2007). The informed consents of the parents were previously authorized by the Review Board of our institution. We didn't conduct research outside our country of residence. We didn't approach the local authorities before beginning work on this study. The full name of the ethics committee of our institution is Comitato di Etica per la Ricerca Scientifica Biomedica, per la Buona Pratica Clinica e per la Sperimentazione dei Farmaci. The parents of the two patients allowed us to publish the descriptive details of their children's malformations.

The parents of the individuals in this manuscript have given written informed consent (as outlined in PLOS consent form) to publish these case details.

### Clinical material

The current study was performed using peripheral blood of the members of the two families treated at the Istituto Giannina Gaslini, Genova, Italy.

#### Cytogenetic, array-CGH and fluorescence*in situ* hybridization analyses

Cytogenetic analysis was performed on GTG-banded metaphases at a resolution of 400–550 bands. Chromosome preparations were made from cultured lymphocytes of patients and their parents.

Fluorescence in situ hybridization (FISH) analysis performed using conventional protocols. BAC clones were selected from the human library RPCI-11 according to the UCSC Genome Browser (http://genome.ucsc.edu/; GRCh37/hg19, February 2009). BAC probes were directly labeled with Spectrum-Orange by nick translation (Vysis/Abbott, Downers Grove, IL).

Array-CGH analyses were performed using the Agilent Human Genome CGH Microarray Kit G3 400 (Agilent Technologies, Palo Alto, USA) with 5.3 Kb overall median probe spacing. Labeling and hybridization were performed following the protocols provided by the manufacturer. Data analysis was performed using the Agilent Genomic Workbench Lite Edition Software 6.5.0.18(2) with the following settings for aberration calling: ADM-2 algorithm (threshold 6) with a moving average of 500 KB and visual inspection of the log2 ratios. DNA sequence information was according to the UCSC Genome Browser (http://genome.ucsc.edu/; GRCh37/hg19, February 2009).

### Expression analysis

For CNP radio-immunoassays, blood samples from the patient, his parents and healthy volunteers were withdrawn in pre-chilled tubes containing aprotinin. After centrifugation at 1.200 rpm for 10 min at 4°C, plasma was collected and stored at −80°C until measurement. Plasma samples were purified on Sep-Pak C18 cartridges (Waters) and CNP content was determined using a RIA kit (Phoenix Pharmaceuticals, Belmont, CA; RKU-012-03) according to the manufacturer's protocol. For analysis of CNP and GAPDH mRNA expression in lymphoblast cells, total RNA was isolated using the RNeasy Mini Kit (Qiagen) according to the manufacturer's protocol. Following DNAse treatment, first strand cDNA was synthesized using the Advantage-RT-for-PCR kit (Clontech). Expression of CNP and GAPDH mRNAs was analyzed by real-time PCR (TaqMan® gene expression master mix; Applied Biosystems) using ABI 7900 HT device.

The following primers were used:

1. NPPC forward: 5′-AGCGTGGGCTCGCCTT-3′
2. NPPC reverse: 5′-CTTGTTGGCTCCTTTGTATTTGC-3′
3. NPPC probe: 5′-6-FAM-TGCAAGAGCACCCCAACGCG-TAMRA- 3′
4. GAPDH forward: 5′-CCACTCCTCCACCTTTGAC -3′
5. GAPDH reverse: 5′-ACCCTGTTGCTGTAGCCA -3′
6. GAPDH probe: 5′-6-FAM-TTGCCCTCAACGACCACTTTGTC-TAMRA- 3′

Reactions included 40 cycles at 96°C for 30 sec and 60°C for 1 min. Sample volume was 20 µl.
